# A Mobile Health Intervention for Self-Management and Lifestyle Change for Persons With Type 2 Diabetes, Part 2: One-Year Results From the Norwegian Randomized Controlled Trial RENEWING HEALTH

**DOI:** 10.2196/mhealth.3882

**Published:** 2014-12-11

**Authors:** Heidi Holmen, Astrid Torbjørnsen, Astrid Klopstad Wahl, Anne Karen Jenum, Milada Cvancarova Småstuen, Eirik Årsand, Lis Ribu

**Affiliations:** ^1^Department of NursingFaculty of Health SciencesOslo and Akershus University College of Applied SciencesOsloNorway; ^2^Department of Health SciencesInstitute of Health and Society, Faculty of MedicineUniversity of OsloOsloNorway; ^3^Department of General PracticeInstitute of Health and Society, Faculty of MedicineUniversity of OsloOsloNorway; ^4^Norwegian Centre for Integrated Care and Telemedicine (NST)University Hospital of North NorwayTromsøNorway

**Keywords:** self-care, mobile applications, cellular phone, telemedicine, counseling, motivational interviewing, diabetes mellitus, type 2, hemoglobin A1c protein, human

## Abstract

**Background:**

Self-management is crucial in the daily management of type 2 diabetes. It has been suggested that mHealth may be an important method for enhancing self-management when delivered in combination with health counseling.

**Objective:**

The objective of this study was to test whether the use of a mobile phone–based self-management system used for 1 year, with or without telephone health counseling by a diabetes specialist nurse for the first 4 months, could improve glycated hemoglobin A_1c_ (HbA_1c_) level, self-management, and health-related quality of life compared with usual care.

**Methods:**

We conducted a 3-arm prospective randomized controlled trial involving 2 intervention groups and 1 control group. Eligible participants were persons with type 2 diabetes with an HbA_1c_ level ≥7.1% (≥54.1 mmol/mol) and aged ≥18 years. Both intervention groups received the mobile phone–based self-management system Few Touch Application (FTA). The FTA consisted of a blood glucose–measuring system with automatic wireless data transfer, diet manual, physical activity registration, and management of personal goals, all recorded and operated using a diabetes diary app on the mobile phone. In addition, one intervention group received health counseling based on behavior change theory and delivered by a diabetes specialist nurse for the first 4 months after randomization. All groups received usual care by their general practitioner. The primary outcome was HbA_1c_ level. Secondary outcomes were self-management (heiQ), health-related quality of life (SF-36), depressive symptoms (CES-D), and lifestyle changes (dietary habits and physical activity). Data were analyzed using univariate methods (*t* test, ANOVA) and multivariate linear and logistic regression.

**Results:**

A total of 151 participants were randomized: 51 to the FTA group, 50 to the FTA-health counseling (FTA-HC) group, and 50 to the control group. Follow-up data after 1 year were available for 120 participants (79%). HbA_1c_ level decreased in all groups, but did not differ between groups after 1 year. The mean change in the heiQ domain skills and technique acquisition was significantly greater in the FTA-HC group after adjusting for age, gender, and education (*P*=.04). Other secondary outcomes did not differ between groups after 1 year. In the FTA group, 39% were substantial users of the app; 34% of the FTA-HC group were substantial users. Those aged ≥63 years used the app more than their younger counterparts did (OR 2.7; 95% CI 1.02-7.12; *P*=.045).

**Conclusions:**

The change in HbA_1c_ level did not differ between groups after the 1-year intervention. Secondary outcomes did not differ between groups except for an increase in the self-management domain of skill and technique acquisition in the FTA-HC group. Older participants used the app more than the younger participants did.

## Introduction

Type 2 diabetes is a complex disease [1,2] with an increasing prevalence worldwide [3,4]. Multifactorial treatment is necessary to improve long-term outcomes as stated in treatment guidelines [1,5,6]. Still, many do not meet the recommended goals for diabetes care [7-9]; in Norway, research has shown that only 20% attain the target for metabolic control for glycated hemoglobin A_1c_ (HbA_1c_), blood pressure, and lipid level, although the quality of care has improved [8]. New treatments are evolving rapidly and self-management is crucial in daily disease management and to prevent macro- and microvascular complications [2,10,11].

The field of technology-supported health care is growing and offers new ways of self-management education and support. Mobile phones are essential in people’s lives today and may serve as a platform for a variety of self-management tools, such as apps. However, the current reviews are inconclusive and the effects of mobile health (mHealth) remain unclear [12-16]. The studies included in these reviews are heterogeneous and have used different mobile phone-based interventions and lengths of follow-up, and people with type 1 and type 2 diabetes are often included in the same studies. In most interventions, patients are monitored by health care personnel in contrast to interventions in which self-management is based on self-monitoring and self-care [14,17]. Despite this, mHealth is recognized as a potential addition to usual care in that some studies have found positive short-term effects on glycemic control, although the effects of the intervention decreased with time [15]. mHealth apps have also been shown to be effective without support from health care personnel, which may reduce health care costs [14].

Apps for mHealth interventions are often combined with health counseling, but the research related to these complex interventions is inconclusive because of heterogeneity in the types of studies [17,18]. Earlier research has shown that phone counseling is feasible, convenient, low cost, and may be an alternative to frequent visits [17,18]. In countries such as Norway, people in rural areas may have less access to specialized health care. A recent Coordination Reform has reorganized the delivery of health care, with more responsibility transferred from specialist health care to primary health care services and with more emphasis on self-management. The application of innovative technologies may be a supplement to this reform [19].

Few studies have used the combination of a mobile phone app for self-management supported by health counseling via telephone. Studies often include monitoring with real-time feedback from health care personnel, which may lead to the investigation of dimensions other than self-management. However, an intervention based largely on the patient’s initiative to self-manage at a frequency that does not interfere with daily life should be feasible in today’s society [20].

Earlier reviews noted the lack of integration of behavior change theory into mHealth research and recommended that interventions should be theory-based [13,17]. Motivational interviewing is a technique in health counseling [21] and a well-known clinical method recommended for use in Norwegian guidelines for persons with diabetes [5]. Research has also indicated an effect of motivational interviewing on persons with type 2 diabetes trying to attain behavior change in lifestyle-related issues [22-24]. Further, some studies have tailored health counseling to the patient’s stage of readiness to change according to the transtheoretical model of stages of change [25] and have demonstrated effects for persons with type 2 diabetes with the use of this model [26,27]. In the present study, both techniques were used in the health counseling.

The current study is the Norwegian part of the European Union collaboration study RENEWING HEALTH (REgioNs of Europe WorkING together for HEALTH), which comprises telehealth interventions in different health care and home settings [28]. The short-term findings after 4 months are described elsewhere [29].

The aim of this study was to determine if the use of a mobile phone–based self-management system for 1 year, with or without telephone health counseling by a diabetes specialist nurse for the first 4 months, could improve HbA_1c_ level, self-management, and health-related quality of life compared with usual care. The primary outcome was glycemic control, as assessed by the HbA_1c_ level. Secondary outcomes were self-management and health-related quality of life, depressive symptoms, and lifestyle changes (dietary habits and physical activity).

## Methods

### Trial Design

We conducted a 3-armed prospective randomized controlled trial (RCT) with a 1:1:1 allocation ratio using block randomization to 1 of 2 intervention groups or to a control group. The allocation has been described in detail elsewhere [30].

### Participants

All participants lived in their homes and received usual care by their general practitioner (GP). They were eligible if they were aged ≥18 years, had an HbA_1c_ level ≥7.1% (54.1 mmol/mol), and were capable of completing questionnaires in the Norwegian language. They also had to be cognitively able to participate and to use the system and devices provided, although prior familiarity with mobile phones was not necessary. The majority of participants were recruited through 2 study centers in the southern and northern parts of Norway in collaboration with their GPs. Some participants were recruited from local public health clinics in the municipalities, through diabetes courses held by the specialist health providers for those newly diagnosed with type 2 diabetes, and through advertisement in The Norwegian Diabetes Association’s media. The HbA_1c_ level was set to HbA_1c_ >7.0% (53 mmol/mol); that is, above the treatment target according to the Norwegian guidelines [5]. Written informed consent was obtained from participants after detailed information about the project was provided by the research team during the start-up meetings. Data collection was obtained through self-reported questionnaires and from medical records at the GPs’ offices. Randomization was performed consecutively.

There were 3 assessment points: baseline (time of randomization) and at 4 and 12 months after randomization. For the follow-up assessment, participants were invited to meet with the research team for data collection (questionnaires). Those not able to attend the follow-up meetings were sent questionnaires and a prepaid envelope to be returned by mail to the study center. All patients were asked to visit their GP for measuring of their HbA_1c_ level and weight at the same time (±14 days) after they had filled in the questionnaires.

### Interventions

#### Overview

The Norwegian study in RENEWING HEALTH was a 1-year intervention to increase self-management comprised of 3 intervention groups: the Few Touch Application (FTA) intervention group, the FTA with health counseling (FTA-HC) intervention group, and the control group [30].

All participants in the 3 groups received usual care by their GP according to national guidelines [5]. This included at least 1 thorough annual visit to their GP for measurement of HbA_1c_ level, blood pressure, blood lipid concentrations, waist circumference, body weight to calculate a body mass index (BMI), screening for late complications, lifestyle advice, and treatment adjustments. Additional visits were recommended to monitor HbA_1c_, fasting glucose, weight, and blood pressure every 2-6 months according to the needs of the patient and to support self-management medical treatment.

#### Control Group

The participants randomized to the control group received usual care [5].

#### Few Touch Application Intervention

In addition to usual care, these participants received a mobile phone with the FTA self-management system. The FTA system provided the user with a diabetes diary app designed to increase self-management through awareness, overview of relevant factors, and motivational feedback through symbols such as smiling faces and color codes in the app [31]. The participants measured blood glucose level with a glucometer (LifeScan OneTouch Ultra Easy), which enabled automatic transfer of the measurement to the diary mobile app through a wireless Bluetooth connection and provided visual graphs, trend reports, and feedback through color coding (below normal, normal, and above normal). The app also consisted of a food habit registration system, a physical activity registration system, a personal goal-setting system, and a general information system. The user entered information about food intake, physical activity, and personal goals manually. Training was in person; a paper manual and a universal serial bus (USB) memory stick with further information were provided to participants. Technical support was available all weekdays between 9 am and 3 pm and was provided by technical staff of the project.

#### Few Touch Application With Health Counseling Intervention

In addition to the mobile phone, FTA system, and usual care, the participants in the FTA-HC group received health counseling for the first 4 months of the project period. The health counseling was based on the transtheoretical model of stages of change [25] and a problem-solving model [32], and used motivational interviewing as a counseling technique [21]. The health counseling in the present study was part of the mHealth intervention. The counseling was delivered as a booster at the start of the intervention. This may have enhanced participants’ identification with the intervention and may have resulted in more autonomous participation and better compliance [22].

**Figure 1:**
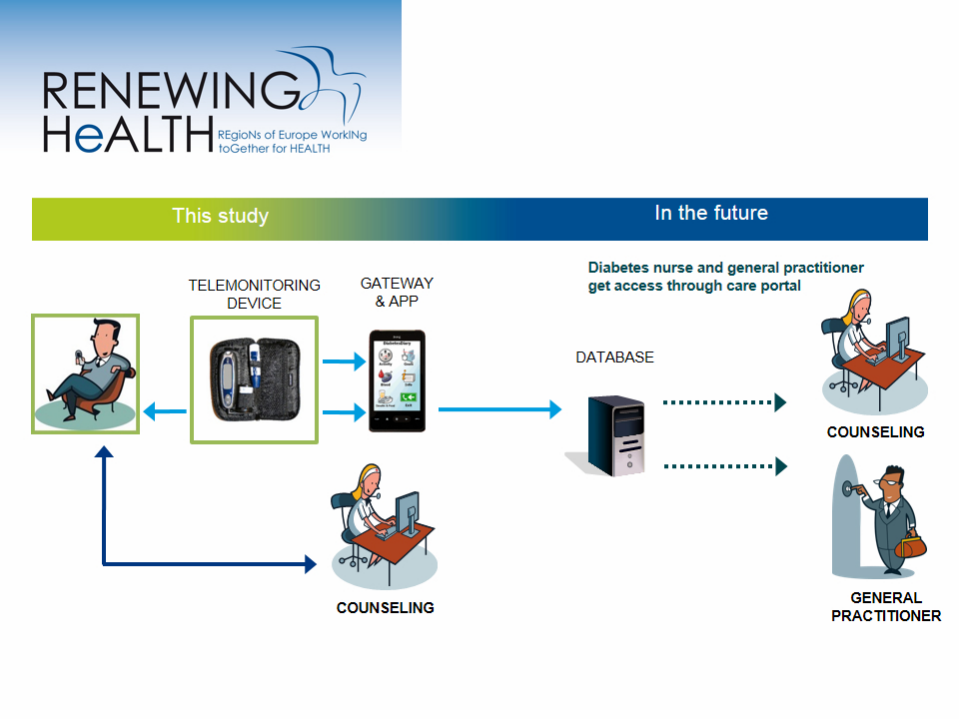
Self-management with the FTA supported by health counseling.

A diabetes specialist nurse delivered the health counseling. She had special training and additional education in diabetes, was supervised by a clinical psychologist, and received support from a dietician when needed. Diet is an important element in the app. The nurse used a client-centered style for enhancing behavior change by helping the patients to explore and resolve ambivalence related to aspects of self-management. We provided a low-intensity intervention with a short counseling duration with few contacts between the patient and health counselor [32]. The counseling was delivered through phone-based conversations each month for 4 months, 5 in total after randomization (with the start-up call), and with no refresher contact thereafter. The calls lasted for 20 minutes (mean) and contained 5 structured modules developed to support self-management and the use of the FTA. The health counseling is described in more detail elsewhere [29,30]. A few days before the call, the diabetes specialist nurse sent a standardized text message through a secure system that allowed the participants to respond or send questions. The plan in the future is that the health personnel get access through their patients’ registrations through a care portal for discussions and increased user participation in treatment (Figure 1).

The participants were recruited to the project because of an HbA_1c_ above the national recommendations (HbA_1c_>7.0%, 53 mmol/mol) [5] and, therefore, they were recommended to measure their blood glucose as a part of their self-management irrespective of insulin use. Most participants not using insulin had been recommended by their GP or diabetes nurse to measure a monthly 24-hour profile of their blood glucose and as such to be aware of their normal blood glucose levels.

Use of the FTA system in GP consultations was an option for the intervention groups; however, the participants had to take the initiative.

### Measures

#### Demographics

Demographic information were self-reported and included age, gender, education, employment status, and cohabitation (including those married and those living with a partner), and are described in detail elsewhere [29,30].

#### Clinical Measures

Clinical characteristics included HbA_1c_, weight, BMI, blood pressure, diabetes duration, comorbidities, complications, medication treatment, hypoglycemia, self-monitoring, and lifestyle variables (smoking, diet, and physical activity). Data were obtained from the GPs or self-reported (diabetes duration, comorbidity, hypoglycemia, self-monitoring, and lifestyle). Of these, only HbA_1c_ and weight were collected at the 1-year follow-up.

#### Primary Outcome

Change in HbA_1c_ level after 1 year was chosen as the primary outcome because it is the main target measure when treating diabetes and is frequently used when evaluating interventions [15]. HbA_1c_ data were collected through the GPs and were assessed primarily with the Siemens DCA Vantage Analyzer a maximum of 2 weeks before or after the follow-up to reduce measurement bias [30,33].

#### Secondary Outcomes

The Health Education Impact Questionnaire (heiQ) [34] was used to assess self-management. This measure contains 40 questions on a 4-level Likert scale, grouped into 8 domains: positive and active engagement in life, health-directed activity, skill and technique acquisition, constructive attitude and approaches, self-monitoring and insight, health service navigation, social integration and support, and emotional well-being. This measure evaluates patient education and self-management interventions for people with chronic conditions. Higher scores reflect greater self-management, except for emotional well-being in which the scale is reversed. The heiQ is a validated measure for evaluating the effectiveness of health education and coping skills, and has been translated into Norwegian and several other languages [34,35].

To evaluate lifestyle and lifestyle changes, we investigated the participants’ dietary habits including recommended food items and traditional Norwegian dietary habits [36], and engagement in physical activity based on intensity, frequency, and duration [37]. The Short-Form 36v2 Health Survey (SF-36) was used to measure overall health-related quality of life [38]. This survey has been translated into Norwegian and validated and tested in a Norwegian setting [39]. Depressive symptoms were measured by the Center for Epidemiologic Studies Depression Scale (CES-D) [40] using a cutoff of ≥16, which indicated that those below the threshold reported no depressive symptoms. For the demographic and clinical measures, a common dataset was provided from the RENEWING HEALTH project administration and data were gathered according to a protocol provided from the project administration [41]. In the analysis, age was dichotomized with a cutoff at ≥63 years, the age of early retirement in Norway. Further details about measures have been published in the study protocol [30].

#### Use of the Few Touch Application

Registrations of the use of the FTA system were collected continuously through automatic data transfer to a secure server and into a usage log. For the FTA-HTC group, further education on usage of the app was supported by the diabetes specialist nurse. A dichotomous variable of substantial or not substantial use of the FTA was made retrospectively based on the usage log. To be categorized as a substantial user, the participant had to be an active user for at least 6 months. An active user was defined as one who had performed ≥5 blood glucose measurements during each of these 6 months and who had ≥50 interactions in the parts of the diary not including collection of data (eg, viewing data or accessing general information).

#### Sample Size

An a priori power calculation indicated that 34 participants in each of the 3 groups would be sufficient to detect significant changes in the primary outcome HbA_1c_ level with an effect size of .35, a significance level of 5%, a standard deviation (SD) of the outcome variable of 0.5, statistical power of 80%, and a 2-tailed significance test. The sample was set to 50 in each of the 3 groups to allow for dropouts and 151 participants were included in total.

### Randomization

Block randomization was performed through the Center of Randomization at the Unit for Applied Clinical Research at the Norwegian University of Science and Technology in Trondheim using the Web Case Report Form.

### Ethics

The Regional Ethics Committee South East approved the protocol and all participants provided written informed consent before randomization.

### Blinding

The study could not be blinded for the participants or GPs and health providers because of the nature of the intervention, which required overt participation [42]. The participants could use the device at visits to their GP as part of usual care. The research team was involved in the assessment of eligibility, data collection, training of patients to use the devices, and follow-up. Thus, those who delivered technical support had to know which group the participants were allocated to.

### Statistical Methods

The baseline characteristics are reported as mean and SD (continuous variables) and counts and percentages (categorical variables). Data not available were considered to be missing and the results were based on intention-to-treat. Baseline differences between groups were assessed with 1-way ANOVA (continuous measurements) and chi square tests (categorical data). Within-group changes were analyzed using Student *t* tests. Multiple linear regression and logistic regression analyses were used to control for possible confounding factors. The final models were adjusted for age, gender, and educational level. Changes in medication (glucose-lowering agents), BMI, depressive symptoms (CES-D), diabetes duration, and comorbidities were added one by one to the final models to investigate the possible confounding effects. When the preceding covariates were not statistically significant, they were not presented in the final model to increase statistical power and precision of our estimates. All tests were 2-sided. *P* values <.05 were considered significant. All analyses were performed using SPSS version 21 (IBM Corp, Armonk, NY, USA).

## Results

### Participant Flow

**Figure 2:**
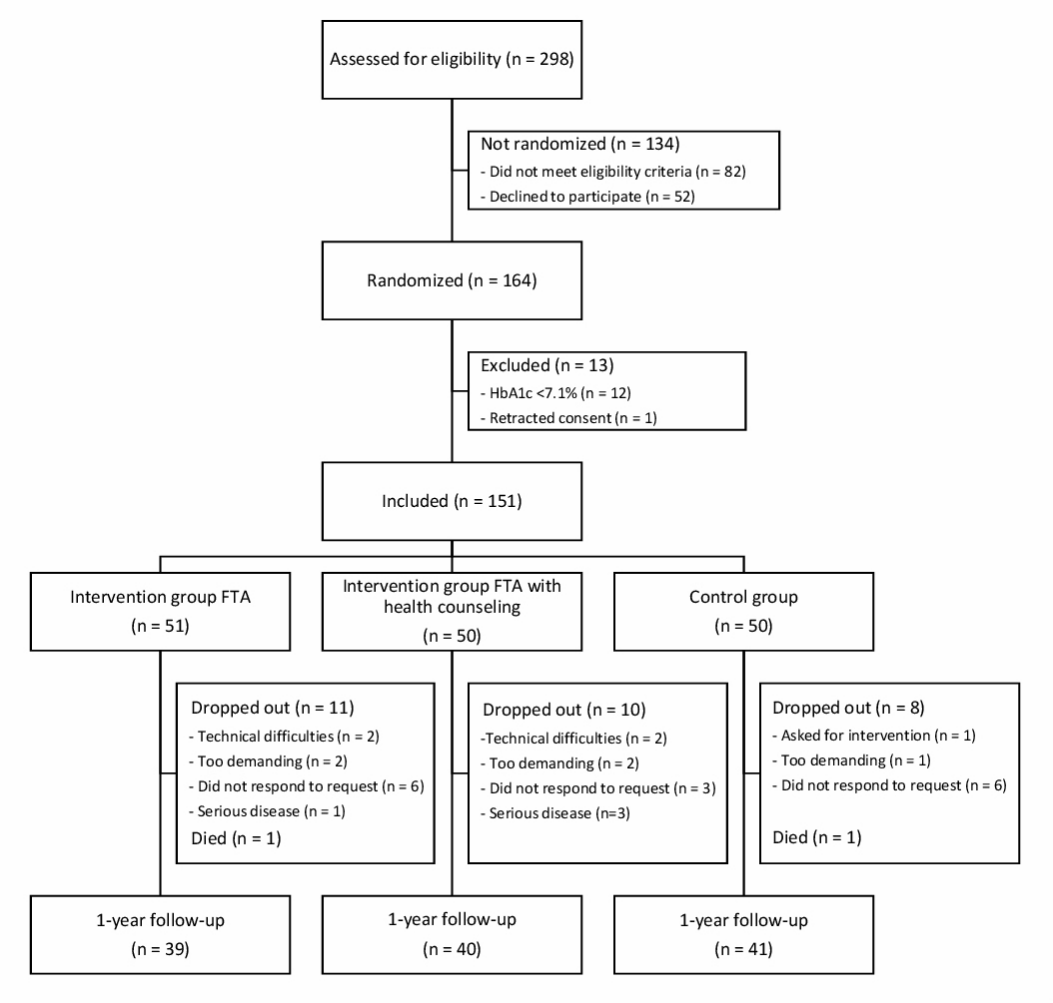
Flowchart of enrollment.

Through the recruitment period, 298 persons were assessed for eligibility; 134 persons were not included, 52 did not wish to participate, and 82 did not meet the eligibility criteria (Figure 2). Of these, 65 had an HbA_1c_ level below the threshold of 7.1% (54.1 mmol/mol), 6 had type 1 diabetes, 4 had interfering comorbidities, and 7 did not fulfill the eligibility criteria for other reasons. Randomization was performed for 164 persons (Figure 1), but 12 were excluded because of an HbA_1c_ level below the 7.1% (54.1 mmol/mol) threshold. One person withdrew consent, leaving a total of 151 participants to be included in the study; 51 were allocated to the FTA intervention, 50 to the FTA-HC intervention, and 50 to the control group.

Inclusion and randomization started in March 2011 and ended in September 2012. The first complete participant dataset was finalized in April 2012 and the follow-up data was finalized in October 2013.

After the 1-year follow-up, there was a total dropout attrition rate of 21% (31/151), with an equal distribution in the groups. Baseline analysis revealed no difference between those lost to follow-up and those who completed the study for all variables. For the primary outcome (HbA_1c_ level), data were obtained for a total of 120 participants after the 1-year follow-up: 39 in the FTA group (dropout attrition 24%, 12/51), 40 in the FTA-HC group (dropout attrition 20%, 10/50), and 41 in the control group (dropout attrition 18%, 9/50). For the secondary self-reported outcomes, data were included from 119 participants, 38 in the FTA group, 40 in the FTA-HC group, and 41 in the control group.

### Demographic and Clinical Characteristics

The demographic and clinical baseline characteristics of the participants have been described in detail elsewhere [29]. Overall, the mean age was 57 years (SD 12), 62 of 151 (41%) were female, and 51 of 151 (34%) had >12 years of education (Table 1). The mean HbA_1c_ level was 8.2% (SD 1.1), 66 mmol/mol (SD 12.3), and the mean BMI was 31.7 kg/m^2^ (SD 6.03). None of the variables listed in the tables differed significantly between groups at baseline. However, a higher proportion of persons in the control group reported depressive symptoms compared with the other 2 groups. The numbers (percentages) of participants whose score exceeded the cutoff value of ≥16 in the CES-D were 17 of 50 (35%) in the control group, 10 of 51 (20%) in the FTA group, and 7 of 50 (14%) in the FTA-HC group (*P*=.04).

**Table 1:** Demographic and clinical characteristics at the baseline (N=151).

Characteristics			N	FTA (n=51)	FTA-HC (n=50)	Control group (n=50)
**Demographics**						
	Age (years), mean (SD)		151	58.6 (11.8)	57.4 (12.1)	55.9 (12.2)
	Gender (female), n (%)		151	17 (33)	25 (50)	20 (40)
	**Education** ^a^ **, n (%)**		151			
		<12 years		26 (51)	26 (52)	31 (62)
		12 years		4 (8)	10 (20)	3 (6)
		>12 years		21 (41)	14 (28)	16 (32)
	**Employment status** ^b^ **, n (%)**		148			
		Employed		22 (44)	31 (63)	26 (53)
		Unemployed		13 (26)	11 (22)	17 (35)
		Retired		15 (30)	7 (14)	6 (12)
	Cohabitation status (cohabiting),^c^ n (%)		151	37 (73)	36 (72)	37 (74)
**Clinical characteristics**						
	**HbA**_**1c**_					
		HbA_1c_ (%), mean (SD)	151	8.1 (1.1)	8.2 (1.1)	8.3 (1.2)
		HbA_1c_ (mmol/mol), mean (SD))	151	65 (12.0)	66 (12.0)	67 (13.1)
		HbA_1c_ (%), median (range)	151	7.8 (7.1-12.4)	7.9 (7.1-11.3)	7.9 (7.1-11.6)
		HbA_1c_ (mmol/mol), median (range)	151	62 (54-112)	63 (54-100)	63 (54-103)
	Weight (kg), mean (SD)		132	98 (23.1)	91 (20.3)	96 (25)
	BMI kg/m^2^, mean (SD)		129	32.4 (6.5)	30.7 (5.6)	32.0 (6.0)
	Systolic blood pressure (mmHg), mean (SD)		121	136 (17.9)	132 (13.7)	134 (14.5)
	Duration of diabetes (years), mean (SD)		138	11.2 (7.3)	9.6 (8.4)	9.4 (5.5)
	**Comorbidities, n (%)**		151			
		0		6 (12)	8 (16)	10 (20)
		1-2		33 (65)	32 (64)	32 (64)
		≥3		12 (23)	10 (20)	8 (16)
	Late complication: foot ulcer, n (%)		151	11 (22)	8 (16)	4 (8)
	Late complication: eye, n (%)		151	7 (14)	3 (6)	9 (18)
**Treatment variables, n (%)**						
	**Glucose-lowering agents, n (%)**		131			
		Diet only		3 (7)	2 (4)	4 (11)
		Oral agents only		20 (44)	27 (57)	16 (42)
		Injections only^d^		9 (20)	7 (15)	3 (8)
		Combination of oral agents and injections		14 (30)	11 (23)	15 (40)
	Hypoglycemia (self-reported), n (%)		148	23 (46)	19 (39)	27 (55)
	Self-monitoring blood glucose, n (%)		151	48 (94)	45 (90)	49 (98)
**Lifestyle variables, n (%)**						
	Smoking (yes)		151	5 (10)	12 (24)	7 (14)
	Physical activity (physically active)^e^		149	18 (37)	16 (32)	17 (34)
	Daily servings of fruit and vegetables		148	2.8 (1.6)	2.9 (1.7)	3.8 (2.7)
	Poultry >3 servings per month		146	33 (67)	26 (52)	28 (60)
	Meat >3 servings per month		143	44 (88)	44 (92)	41 (91)
	Fish >3 servings per month		148	41 (82)	38 (78)	37 (76)

^a^ Education: some high school or less (<12 years), high school graduate (12 years), or some college or more (>12 years).

^b^ Employment status: employed (state employee, private employee, self-employed, or employed part-time); unemployed (student, military duty, homemaker, unemployed, or unable to work); and retired.

^c^ Cohabitation status: living alone (not married, divorced, separated, or widowed); and cohabiting (married or living with someone).

^d^ Injections were both insulin and other blood glucose–lowering injections.

^e^ Physically active: those with >60 min per week at an intensity of “being short of breath” or higher intensity.

### Primary Outcome Measure: HbA_1c_ Level

**Figure 3:**
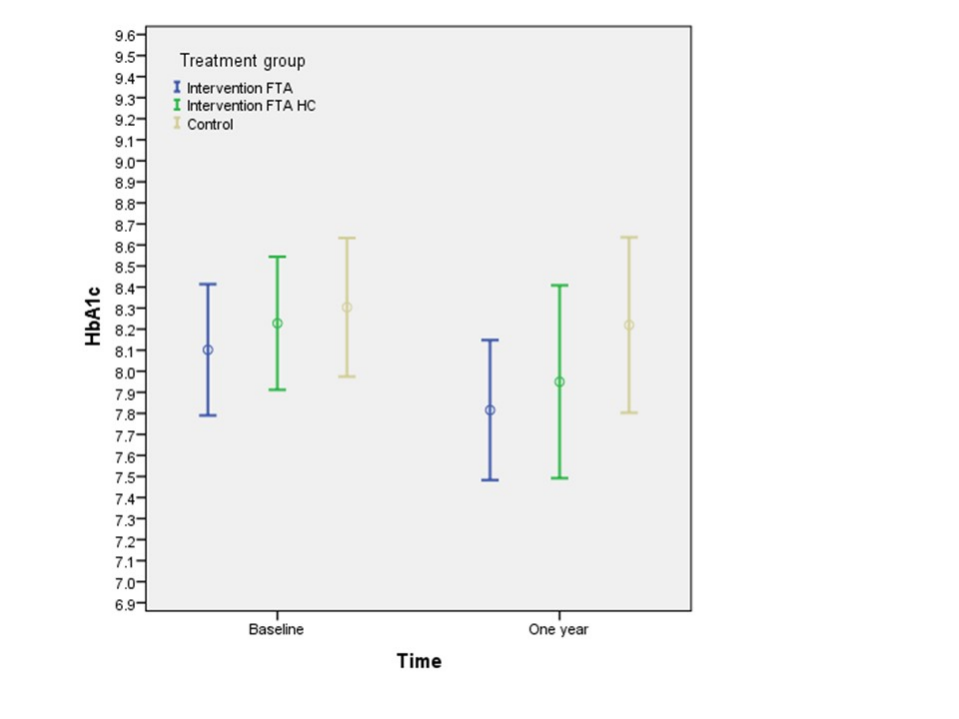
Mean HbA1c levels (95% CI) at baseline and 1-year follow-up (N=119).

The change in HbA_1c_ level did not differ significantly between the 3 groups after 1 year. However, HbA_1c_ level declined within all groups and none of the participants in any of the groups reached their pretest levels at the 1-year follow-up (Figure 3).

Adjusting for age, gender, and educational level did not affect the change in HbA_1c_ level nor did inclusion of possible confounders, such as changes in medication (glucose-lowering agents), BMI, depressive symptoms (CES-D), diabetes duration, and comorbidities (Table 2).

**Table 2:** Mean HbA_1c_ level, body weight, and heiQ domains at baseline and 1-year follow-up, and changes for those with 2 measurements.

Variables by group		n	Baseline, mean (95% CI)	1-year follow-up, mean (95% CI)	Change, mean (95% CI)
**HbA_1c_ (%)**					
	FTA	39	8.1 (7.72, 8.53)	7.8 (7.48, 8.15)	–0.31 (–0.67, 0.05)
	FTA-HC	40	8.1 (7.76, 8.43)	8.0 (7.49, 8.41)	–0.15 (–0.58, 0.29)
	Control	41	8.4 (7.97, 8.76)	8.2 (7.77, 8.61)	–0.16 (–0.50, 0.18)
**HbA_1c_ (mmol/mol)**					
	FTA	39	65 (61,70)	62 (58,66)	–3.4 (–7.4,0.6)
	FTA-HC	40	65 (61,69)	63 (58,68)	–1.6 (–6.3,3.1)
	Control	41	68 (64,72)	66 (62,71)	–1.7 (–5.4,2.0)
**Weight (kg)**					
	FTA	33	96.3 (87.99, 104.64)	95.0 (87.54, 103.22)	–1.3 (–3.05, 0.43)
	FTA-HC	34	89.7 (82.45, 96.90)	88.9 (82.28, 95.67)	–0.7 (–2.29, 0.84)
	Control	36	94.3 (85.31, 103.22)	93.0 (84.44, 101.36)	–1.2 (–2.75, 0.54)
**Positive and active engagement in life**					
	FTA	38	3.23 (3.08, 3.38)	3.19 (3.04, 3.34)	–0.04 (–0.18, 0.09)
	FTA-HC	40	3.20 (3.08, 3.31)	3.22 (3.08, 3.36)	0.02 (–0.15, 0.19)
	Control	41	3.12 (2.95, 3.29)	3.09 (2.94, 3.24)	–0.03 (–0.19, 0.13)
**Health-directed activity**					
	FTA	38	2.78 (2.52, 3.04)	2.82 (2.60, 3.05)	0.04 (–0.16, 0.25)
	FTA-HC	40	2.78 (2.57, 2.99)	2.81 (2.57, 3.04)	0.03 (–0.16, 0.21)
	Control	41	2.71 (2.51, 2.92)	2.81 (2.58, 3.04)	0.10 (–0.08, 0.27)
**Skill and technique acquisition**					
	FTA	38	2.92 (2.79, 3.04)	2.88 (2.69, 3.06)	–0.04 (–0.20, 0.12)
	FTA-HC	40	2.89 (2.75, 3.02)	3.08 (2.96, 3.21)	0.19 (0.05, 0.33)^a^
	Control	41	2.95 (2.83, 3.06)	2.94 (2.77, 3.12)	–0.01 (–0.14, 0.13)
**Constructive attitudes and approaches**					
	FTA	38	3.17 (2.98, 3.36)	3.13 (3.00, 3.26)	–0.04 (–0.21, 0.13)
	FTA-HC	40	3.23 (3.09, 3.38)	3.33 (3.19, 3.47)	0.10 (–0.02, 0.21)
	Control	41	3.19 (3.02, 3.36)	3.19 (3.02, 3.36)	0.00 (–0.13, 0.13)
**Self-monitoring and insight**					
	FTA	38	3.06 (2.95, 3.15)	3.09 (2.98, 3.19)	0.04 (–0.07, 0.15)
	FTA-HC	40	3.09 (2.99, 3.18)	3.18 (3.06, 3.30)	0.09 (–0.01, 0.19)
	Control	41	3.14 (3.03, 3.24)	3.15 (3.02, 3.28)	0.01 (–0.12, 0.13)
**Health service navigation**					
	FTA	38	3.14 (2.97, 3.31)	3.03 (2.86, 3.20)	–0.11 (–0.25, 0.04)
	FTA-HC	40	3.06 (2.91, 3.20)	3.14 (2.96, 3.31)	0.08 (–0.03, 0.20)
	Control	41	3.16 (3.00, 3.33)	3.27 (3.09, 3.44)	0.11 (–0.05, 0.26)
**Social integration and support**					
	FTA	38	3.04 (2.87, 3.21)	2.93 (2.77, 3.09)	–0.11 (–0.23, 0.02)
	FTA-HC	40	3.02 (2.86, 3.17)	3.02 (2.86, 3.19)	0.01 (–0.09, 0.11)
	Control	41	2.94 (2.74, 3.15)	2.95 (2.74, 3.16)	0.01 (–0.14, 0.16)
**Emotional well-being**					
	FTA	38	2.99 (2.77, 3.20)	2.98 (2.76, 3.20)	–0.01 (–0.16, 0.13)
	FTA-HC	40	2.99 (2.81, 3.17)	3.04 (2.84, 3.25)	0.05 (–0.12, 0.22)
	Control	41	2.81 (2.57, 3.05)	2.87 (2.64, 3.11)	0.07 (–0.11, 0.24)

^a^ Change was statistically significant (*P*<.05).

### Secondary Outcome Measures

#### Weight

Body weight was slightly reduced in all 3 groups at the 1-year follow-up, although not significant (Table 2). However, the change in weight did not differ between groups at the 1-year follow-up.

#### Health Education Impact Questionnaire

Table 2 presents the mean scores for the 8 heiQ domains and the mean changes at the 1-year follow-up. Except for skill and technique acquisition in the FTA-HC group, no statistically significant changes were found between groups. However, as shown in Table 3, there were significant differences in the changes in 1 of the 8 domains between the control group and 1 of the intervention groups.

**Table 3:** Changes in HbA_1c_ level, skill and technique acquisition, and health service navigation for the intervention groups versus the control group, unadjusted and adjusted for age, gender, and educational level in multiple linear regression analysis.^a^

Group		Unadjusted B	95% CI	*P*	Adjusted B^a^	95% CI	*P*
**HbA_1c_ (%)**							
	FTA	–0.15	–0.68, 0.37	.57	–0.22	–0.75, 0.32	.42
	FTA-HC	0.01	–0.51, 0.53	.97	0.01	–0.52, 0.54	.97
	Control (ref)						
**HbA_1c_ (mmol/mol)**							
	FTA	–1.7	–7.4, 4.1		–2.4	–8.2, 3.5	.42
	FTA-HC	0.1	–5.6, 5.8		0.1	–5.6, 5.9	.97
	Control (ref)						
**Skill and technique acquisition**							
	FTA	–0.04	–0.24, 0.16	.71	–0.03	–0.22, 0.17	.79
	FTA-HC	0.20	0.004, 0.40	.046	0.21	0.01, 0.40	.04
	Control (ref)						
**Health service navigation**							
	FTA	–0.21	–0.41,–0.02	.03	–0.19	–0.38, 0.01	.06
	FTA-HC	–0.02	–0.21, 0.17	.82	–0.004	–0.19, 0.19	.97
	Control (ref)						

^a^ This table presents 3 final multiple linear regression models, all adjusted for age, gender, and education.

After adjusting for age, gender, and educational level, the mean change in skill and technique acquisition was still significantly higher in the FTA-HC group (B=0.21; 95% CI 0.01-0.40; *P*=.04). The mean change in health service navigation was significantly smaller in the FTA group before but not after adjusting for age, gender, and educational level (B=–0.19; CI –0.38 to 0.01; *P*=.06) compared with the control group.

When analyzing the effect of depressive symptoms independently of group allocation, we found that those who reported depressive symptoms (CES-D score ≥16 at baseline, indicating more depressive symptoms) reported a higher change in heiQ than those who did not report such symptoms. Both analyses of change in heiQ after 1 year were adjusted for age and gender. In the domains of positive and active engagement in life, the results were B=0.24, (95% CI 0.01-0.46; (*P*=.04) and for social integration and support were B=0.22 (95% CI 0.03-0.41; *P*=.02).

#### Health-Related Quality of Life and Depressive Symptoms

There were no significant differences in any of the 8 subscales or in the 2 summary component scores of the SF-36 between the 3 groups at the 1-year follow-up in both the unadjusted and adjusted analyses. The change in depressive symptoms measured with the CES-D did not differ significantly between groups for the total score (continuous variable) or for the number/percentage of participants with a score greater than the cutoff of ≥16 both before and after adjustments.

#### Changes in Reported Physical Activity and Nutritional Habits

There were no significant differences between the groups in self-reported levels of physical activity (inactive to active or opposite). The changes in the intake of fruits and vegetables, meat, chocolate, and fish after 1 year did not differ between the 3 groups (results not shown).

#### Use of the Few Touch Application and Health Counseling

Of those randomized to the FTA group, 20 of 51 (39%) were categorized as substantial users. In the FTA-HC group, 17 of 50 (34%) used the FTA part of the intervention substantially, and all these people attended ≥4 health counseling sessions; 42 of 50 (84%) attended ≥4 sessions of health counseling regardless of their FTA use.

Analyses of substantial versus nonsubstantial users of only the FTA, regardless of the intervention groups, did not reveal any statistically significant differences between groups regarding SF-36, heiQ, or depressive symptoms (CES-D). However, participants aged ≥63 years were more likely to be substantial users of the app (OR 2.7; 95% CI 1.02-7.12; *P*=.045) compared with younger participants.

### Adverse Events

No serious adverse clinical events were reported from enrollment to the 1-year follow-up. However, a few undesired technical events were reported, such as trouble with the Bluetooth pairing required for automatic transmission of data from the glucometer to the app in the mobile phone. This may have been stressful for those affected and has been shown to lead to less satisfaction and decreased use of the technology in a previous study [13]. The project could not pay for mobile use if the participants were traveling abroad and some participants experienced high mobile costs for use of the mobile phone app in other countries (because of different rates for different network operators). However, we did inform all participants of this risk before they entered the trial.

## Discussion

Although HbA_1c_ level declined in all groups, the change did not differ significantly between either of the intervention groups and the control group after 1 year. However, the mean HbA_1c_ level did not increase to the baseline level in any of the 3 groups. We found no effects on secondary outcomes other than a significant positive change in self-management reflected by the skill and technique acquisition scale in the FTA-HC group. Interestingly, participants aged ≥63 years were more likely to use the app.

In this study, we conducted a low-intensity mHealth intervention based on self-management with a mobile app and with a health-counseling booster for the first 4 months in one of the intervention groups. Previous reviews have investigated follow-up and intervention duration, and have found a trend of decreasing intervention effect over time [15,18]. Although interest in mHealth interventions may decrease over time [17,43], it has been shown previously that regular contact with clinical practice may improve glycemic control [15,16] and positive outcomes in general [17]. The participants in our study had only the health counseling intervention in one of the intervention groups at the beginning of the study and a more intense intervention during the 1-year follow-up or booster appointments could have strengthened their self-management and behavior change.

The finding that the FTA-HC intervention group tended to have a greater change in self-management, as shown by the increase in skill and technique acquisition, may mean that they had an increased ability to reduce their symptoms related to type 2 diabetes and to manage their health effectively, including greater skills for using technical aids. A lack of effect in the other domains of self-management could indicate that our intervention did not reach those at highest risk of a decline in health [44]. The degree of self-management may be less in people with type 2 diabetes compared with those with type 1 diabetes because of the intensity of treatment and need for self-measuring of blood glucose levels by those who are insulin dependent [15]. However, some type 2 diabetes insulin users are also in need of a similar self-management intensity. Reviews are inconsistent about whether mHealth is more effective in people with type 1 or type 2 diabetes [15,20]. Most of our participants reported that they were self-monitoring their blood glucose level at the start of the study, suggesting that they were already self-managing at some level irrespective of insulin use.

The HbA_1c_ level is widely used for evaluation of interventions, but its relevance to self-management has been questioned in the past few years [15,18] because the focus on glycemic control may not always reflect the degree of self-management. To date, few mHealth studies evaluating self-management have included a self-management outcome with appropriate measures [35,45]. The choice of outcome measures is critical. The emphasis in the present study is on self-management and the primary outcome, HbA_1c_, may not reflect the relevant self-management outcomes for the participants. In this study, we found that many participants did not know their HbA_1c_ level at enrollment and many had a too low HbA_1c_ to be included.

Interventions are often designed without sufficient knowledge about the target group and without a theoretical framework [46]. Although this study used both theory and thorough analyses of the literature beforehand, more research about how to design and implement behavior change interventions is needed. An interesting framework has been developed with a behavior change model with essential conditions such as capability, opportunity, and motivation, including intervention strategies addressing these conditions specifically [46]. If a self-management intervention should improve HbA_1c_, it must first effectively improve healthy eating, physical activity, and adherence to medication. Therefore, we need to know how we can support and effectively motivate a person’s readiness for behavior change. Future research must include the users as part of the team when developing appropriate interventions tailored to their needs [11,46,47].

Lack of findings in many behavior change studies may also relate to a lack of key components in available apps for persons with type 2 diabetes. Apps should be designed in the context of the current guidelines for treatment of type 2 diabetes to increase self-management [12,13]. It has been shown previously how integrated daily use is more likely if the self-management components are offered in a mobile phone app, and electronic diaries are thought to improve self-management [48], as in this study. Further, solutions are provided to reduce the potential for erroneous imputations for functions such as transfer of blood glucose data [12,13]. However, the perceived benefits must outweigh the effort of using the app, especially because self-management is an ongoing process that requires many iterations every day [2]. The most frequent component offered in mobile phone apps is blood glucose measurement, but education in self-monitoring of blood glucose [12] and in the use of the application [13,15] is often lacking.

There are also other possible explanations for the lack of difference in the change in HbA_1c_ levels between groups. A total 39% of participants were substantial users of the app during the 1-year follow-up. The lack of effects on predefined outcomes may also relate to low use of the FTA, partly caused by outdated technology at the end of the study. The actual use of a mHealth intervention may reflect the external validity better than does the rate of dropouts [43]. In this study, attrition occurred in participants who did not use the intervention or used it infrequently. The common limit for threatened external validity is a 20% dropout rate [49], but high dropout attrition is expected in trials investigating innovative technology because of technical difficulties and cumbersome user interfaces. Our attrition rates are relatively small in comparison with others [43].

Traditionally, the RCT is the gold standard for clinical trials. In this study, we achieved successful randomization with no statistically significant differences between the 3 groups at baseline. Moreover, all patients were recruited from the primary health care system, which may increase the generalizability of our results [18]. During this study, new and improved versions of mobile phones hit the market and participants reported this as the reason for some of the cases of low use of the mobile phones given to the participants. Outdated equipment may be a problem when using RCTs for testing mobile interventions because of the often-prolonged inclusion process. In future research within the digital area, we should consider other designs and evaluation methods that have a shorter turnover than RCTs.

Some of the results were unexpected, such as the increased use among the older participants (aged ≥63 years). In previous research, a lack of effect was attributed to a fear of technology with increasing age [14], although others have suggested that compliance may be higher in older people [20]. Our findings suggest that age may not be the barrier that many expect. Generalization of the results of this single trial must be made with caution because of the participants’ motivation and preferences for entering the study. It is preferable that the characteristics of those interested in mHealth interventions in the target population should be investigated before the study starts [50].

In summary, we have successfully conducted a low-intensity RCT to test a mobile diabetes self-management system with and without health counseling. There were no significant differences in the change in HbA_1c_ between the intervention groups and the control group. Skill and technique acquisition increased in those who received health counseling in addition to the self-management app. This may be important to their daily self-management of diabetes. Our findings indicate that age may not hinder the use of technology, as suggested by earlier research, but further research is needed to confirm this finding.

## Abbreviations

BMI: body mass index
CES-D: Center for Epidemiologic Studies Depression Scale
FTA: Few Touch Application
FTA-HC: FTA with health counseling
GP: general practitioner
HbA1c: glycated hemoglobin A1c
heiQ: Health Education Impact Questionnaire
mHealth: mobile health
RENEWING HEALTH: REgioNs of Europe WorkING together for HEALTH
SF-36: Short-Form 36v2 Health Survey

## References

[1] Inzucchi SE, Bergenstal RM, Buse JB, Diamant M, Ferrannini E, Nauck M, Peters AL, Tsapas A, Wender R, Matthews DR (2012). Management of hyperglycaemia in type 2 diabetes: a patient-centered approach. Position statement of the American Diabetes Association (ADA) and the European Association for the Study of Diabetes (EASD). Diabetologia.

[2] Marrero DG, Ard J, Delamater AM, Peragallo-Dittko V, Mayer-Davis EJ, Nwankwo R, Fisher EB (2013). Twenty-first century behavioral medicine: a context for empowering clinicians and patients with diabetes: a consensus report. Diabetes Care.

[3] Strøm H, Selmer R, Birkeland KI, Schirmer H, Berg TJ, Jenum AK, Midthjell K, Berg C, Stene LC (2014). No increase in new users of blood glucose-lowering drugs in Norway 2006-2011: a nationwide prescription database study. BMC Public Health.

[4] Shaw JE, Sicree RA, Zimmet PZ (2010). Global estimates of the prevalence of diabetes for 2010 and 2030. Diabetes Res Clin Pract.

[5] Claudi T (2009). Oslo: Helsedirektoratet. IS-.

[6] American Diabetes Association (2014). Standards of medical care in diabetes--2014. Diabetes Care.

[7] Ali MK, Bullard KM, Saaddine JB, Cowie CC, Imperatore G, Gregg EW (2013). Achievement of goals in U.S. diabetes care, 1999-2010. N Engl J Med.

[8] Claudi T, Ingskog W, Cooper JG, Jenum AK, Hausken MF (2008). [Quality of diabetes care in Norwegian general practice]. Tidsskr Nor Laegeforen.

[9] de Pablos-Velasco P, Parhofer KG, Bradley C, Eschwège E, Gönder-Frederick L, Maheux P, Wood I, Simon D (2014). Current level of glycaemic control and its associated factors in patients with type 2 diabetes across Europe: data from the PANORAMA study. Clin Endocrinol (Oxf).

[10] Norris SL, Lau J, Smith SJ, Schmid CH, Engelgau MM (2002). Self-management education for adults with type 2 diabetes: a meta-analysis of the effect on glycemic control. Diabetes Care.

[11] Haas L, Maryniuk M, Beck J, Cox CE, Duker P, Edwards L, Fisher EB, Hanson L, Kent D, Kolb L, McLaughlin S, Orzeck E, Piette JD, Rhinehart AS, Rothman R, Sklaroff S, Tomky D, Youssef G, 2012 Standards Revision Task Force (2014). National standards for diabetes self-management education and support. Diabetes Care.

[12] Chomutare T, Fernandez-Luque L, Arsand E, Hartvigsen G (2011). Features of mobile diabetes applications: review of the literature and analysis of current applications compared against evidence-based guidelines. J Med Internet Res.

[13] El-Gayar O, Timsina P, Nawar N, Eid W (2013). Mobile applications for diabetes self-management: status and potential. J Diabetes Sci Technol.

[14] Baron J, McBain H, Newman S (2012). The impact of mobile monitoring technologies on glycosylated hemoglobin in diabetes: a systematic review. J Diabetes Sci Technol.

[15] Marcolino MS, Maia JX, Alkmim MB, Boersma E, Ribeiro AL (2013). Telemedicine application in the care of diabetes patients: systematic review and meta-analysis. PLoS One.

[16] Tao D, Or CK (2013). Effects of self-management health information technology on glycaemic control for patients with diabetes: a meta-analysis of randomized controlled trials. J Telemed Telecare.

[17] Fitzner K, Moss G (2013). Telehealth--an effective delivery method for diabetes self-management education?. Popul Health Manag.

[18] Cassimatis M, Kavanagh DJ (2012). Effects of type 2 diabetes behavioural telehealth interventions on glycaemic control and adherence: a systematic review. J Telemed Telecare.

[19] Norwegian Ministry of Health and Care Services Summary in English: Report No 47 (2008-2009) to the Storting.

[20] Liang X, Wang Q, Yang X, Cao J, Chen J, Mo X, Huang J, Wang L, Gu D (2011). Effect of mobile phone intervention for diabetes on glycaemic control: a meta-analysis. Diabet Med.

[21] Rollnick S, Miller WR, Butler CC (2008). Motivational Interviewing in Health Care: Helping Patients Change Behavior (Applications of Motivational Interviewing).

[22] Rubak S, Sandbaek A, Lauritzen T, Borch-Johnsen K, Christensen B (2009). General practitioners trained in motivational interviewing can positively affect the attitude to behaviour change in people with type 2 diabetes. One year follow-up of an RCT, ADDITION Denmark. Scand J Prim Health Care.

[23] Söderlund LL, Madson MB, Rubak S, Nilsen P (2011). A systematic review of motivational interviewing training for general health care practitioners. Patient Educ Couns.

[24] Lundahl B, Moleni T, Burke BL, Butters R, Tollefson D, Butler C, Rollnick S (2013). Motivational interviewing in medical care settings: a systematic review and meta-analysis of randomized controlled trials. Patient Educ Couns.

[25] Prochaska JO, DiClemente CC, Norcross JC (1992). In search of how people change. Applications to addictive behaviors. Am Psychol.

[26] Ruggiero LP, Prochaska JO (1993). Readiness for change: application of the transtheoretical model to diabetes. Diabetes Spectrum.

[27] Andrés A, Gómez J, Saldaña C (2008). Challenges and applications of the transtheoretical model in patients with diabetes mellitus. Disease Management and Health Outcomes.

[28] RenewingHealth.

[29] Torbjørnsen A, Jenum AK, Småstuen MC, Årsand E, Holmen H, Wahl AK, Ribu LA (2014). Mobile health intervention with and without health counseling for persons with type 2 diabetes: Results from a randomized controlled trial in the Norwegian part of RENEWING HEALTH Part 1. JMIR mHealth uHealth.

[30] Ribu L, Holmen H, Torbjørnsen A, Wahl AK, Grøttland A, Småstuen MC, Elind E, Bergmo TS, Breivik E, Arsand E (2013). Low-intensity self-management intervention for persons with type 2 diabetes using a mobile phone-based diabetes diary, with and without health counseling and motivational interviewing: protocol for a randomized controlled trial. JMIR Res Protoc.

[31] Arsand E, Tatara N, Østengen G, Hartvigsen G (2010). Mobile phone-based self-management tools for type 2 diabetes: the few touch application. J Diabetes Sci Technol.

[32] Richards D, Chellingsworth M, Hope R, Turpin G, Whyte M (2010). Reach Out: National Programme Supervisor Materials to Support the Delivery of Training for Psychological Wellbeing Practitioners Delivering Low Intensity Interventions.

[33] McNamara R, Robling M, Hood K, Bennert K, Channon S, Cohen D, Crowne E, Hambly H, Hawthorne K, Longo M, Lowes L, Playle R, Rollnick S, Gregory JW (2010). Development and Evaluation of a Psychosocial Intervention for Children and Teenagers Experiencing Diabetes (DEPICTED): a protocol for a cluster randomised controlled trial of the effectiveness of a communication skills training programme for healthcare professionals working with young people with type 1 diabetes. BMC Health Serv Res.

[34] Osborne RH, Elsworth GR, Whitfield K (2007). The Health Education Impact Questionnaire (heiQ): an outcomes and evaluation measure for patient education and self-management interventions for people with chronic conditions. Patient Educ Couns.

[35] Schuler M, Musekamp G, Faller H, Ehlebracht-König I, Gutenbrunner C, Kirchhof R, Bengel J, Nolte S, Osborne RH, Schwarze M (2013). Assessment of proximal outcomes of self-management programs: translation and psychometric evaluation of a German version of the Health Education Impact Questionnaire (heiQ™). Qual Life Res.

[36] Larsen IK, Grotmol T, Almendingen K, Hoff G (2007). Impact of colorectal cancer screening on future lifestyle choices: a three-year randomized controlled trial. Clin Gastroenterol Hepatol.

[37] Kurtze N, Rangul V, Hustvedt BE, Flanders WD (2007). Reliability and validity of self-reported physical activity in the Nord-Trøndelag Health Study (HUNT 2). Eur J Epidemiol.

[38] Ware JE (2000). SF-36 health survey update. Spine (Phila Pa 1976).

[39] Loge JH, Kaasa S, Hjermstad MJ, Kvien TK (1998). Translation and performance of the Norwegian SF-36 Health Survey in patients with rheumatoid arthritis. I. Data quality, scaling assumptions, reliability, and construct validity. J Clin Epidemiol.

[40] Radloff, LS (1977). The CES-D Scale: A Self-Report Depression Scale for Research in the General Population. Applied Psychological Measurement.

[41] RenewingHealth.

[42] Baker TB, Gustafson DH, Shaw B, Hawkins R, Pingree S, Roberts L, Strecher V (2010). Relevance of CONSORT reporting criteria for research on eHealth interventions. Patient Educ Couns.

[43] Eysenbach G (2005). The law of attrition. J Med Internet Res.

[44] Osborne RH, Batterham R, Livingston J (2011). The evaluation of chronic disease self-management support across settings: the international experience of the health education impact questionnaire quality monitoring system. Nurs Clin North Am.

[45] Glasgow RE, Peeples M, Skovlund SE (2008). Where is the patient in diabetes performance measures? The case for including patient-centered and self-management measures. Diabetes Care.

[46] Michie S, van Stralen MM, West R (2011). The behaviour change wheel: a new method for characterising and designing behaviour change interventions. Implement Sci.

[47] Verhoeven F, Tanja-Dijkstra K, Nijland N, Eysenbach G, van Gemert-Pijnen L (2010). Asynchronous and synchronous teleconsultation for diabetes care: a systematic literature review. J Diabetes Sci Technol.

[48] Vuong AM, Huber JC, Bolin JN, Ory MG, Moudouni DM, Helduser J, Begaye D, Bonner TJ, Forjuoh SN (2012). Factors affecting acceptability and usability of technological approaches to diabetes self-management: a case study. Diabetes Technol Ther.

[49] Altman D (1991). G. Practical Statistics for Medical Research.

[50] Buysse H, De Moor G, Coorevits P, Van Maele G, Kaufman J, Ruige JA (2011). Main characteristics of type 1 and type 2 diabetic patients interested in the use of a telemonitoring platform. J Nurs Healthc Chronic Illn.

